# Culling-Induced Changes in Badger (*Meles meles*) Behaviour, Social Organisation and the Epidemiology of Bovine Tuberculosis

**DOI:** 10.1371/journal.pone.0028904

**Published:** 2011-12-14

**Authors:** Philip Riordan, Richard John Delahay, Chris Cheeseman, Paul James Johnson, David Whyte Macdonald

**Affiliations:** 1 Wildlife Conservation Research Unit, Department of Zoology, University of Oxford, Oxford, United Kingdom; 2 The Food and Environment Research Agency, York, United Kingdom; Stanford University, United States of America

## Abstract

In the UK, attempts since the 1970s to control the incidence of bovine tuberculosis (bTB) in cattle by culling a wildlife host, the European badger (*Meles meles*), have produced equivocal results. Culling-induced social perturbation of badger populations may lead to unexpected outcomes. We test predictions from the ‘perturbation hypothesis’, determining the impact of culling operations on badger populations, movement of surviving individuals and the influence on the epidemiology of bTB in badgers using data dervied from two study areas within the UK Government's Randomised Badger Culling Trial (RBCT). Culling operations did not remove all individuals from setts, with between 34–43% of badgers removed from targeted social groups. After culling, bTB prevalence increased in badger social groups neighbouring removals, particularly amongst cubs. Seventy individual adult badgers were fitted with radio-collars, yielding 8,311 locational fixes from both sites between November 2001 and December 2003. Home range areas of animals surviving within removed groups increased by 43.5% in response to culling. Overlap between summer ranges of individuals from Neighbouring social groups in the treatment population increased by 73.3% in response to culling. The movement rate of individuals between social groups was low, but increased after culling, in Removed and Neighbouring social groups. Increased bTB prevalence in Neighbouring groups was associated with badger movements both into and out of these groups, although none of the moving individuals themselves tested positive for bTB. Significant increases in both the frequency of individual badger movements between groups and the emergence of bTB were observed in response to culling. However, no direct evidence was found to link the two phenomena. We hypothesise that the social disruption caused by culling may not only increase direct contact and thus disease transmission between surviving badgers, but may also increase social stress within the surviving population, causing immunosuppression and enhancing the expression of disease.

## Introduction

Attempts to manage infectious diseases in wildlife populations have varying consequences for the ecology of the target populations, depending on the methods used [1], [2]. Culling has frequently been used in attempts to control wildlife disease, based on the assumptions that transmission is frequency or density dependent and that there is a population density threshold below which the disease cannot persist [3], [4]. Ecological and behavioural complexities in the host species' response to incomplete culling may alter the outcome of such control as a result of social perturbation [5]–[7].

Epidemiological patterns of directly transmitted infectious diseases are the product of contacts between individuals that permit disease transmission [8], and heterogeneous mixing of individuals is of particular importance [9]. Also, management interventions to control infectious disease in wildlife populations may themselves influence individual behaviours, such as dispersal, which may in turn result in counter-productive outcomes such as further disease spread [5], [7], [10]. Predicting such outcomes in wild animal populations is enormously challenging, not least because the behavioural processes underlying disease transmission are notoriously difficult to study [11].

*Mycobacterium bovis* (the causative agent of bovine tuberculosis; bTB), can infect a wide range of mammals [12]. Since the early 1970s in the UK, the European badger (*Meles meles*) has been implicated as a reservoir for bTB in cattle [13]. Since then, badgers have been culled as part of attempts to control the disease in cattle, although the incidence of bTB in UK cattle herds has continued to increase [14]–[16]; although the effects of previous culling policies have been difficult to determine, since they were not carried out as scientific trials [14]. In 1998, the Randomised Badger Culling Trial (RBCT), was initiated to quantify the impact of badger culling on cattle herd breakdown rates [15], following the recommendations of a UK Government review [14].

Undisturbed, moderate to high density badger populations in lowland England are often organised into relatively stable, mixed-sex social groups [17], [18]. Evidence from field studies suggests that this pattern of social organisation mitigates the spread of bTB between groups, and that disease transmission rates between social groups are lower than those within groups [19], [20]. The perturbation hypothesis predicts that culling may affect social behaviour in ways that alter bTB epidemiology among the survivors [4], [7], [21], [22]. The effects of culling induced perturbation might take various forms, and in the case of badgers [7], [23] might alter the rate of inter-group disease transmission, by for example changing rates of inter-group movements and interactions [24] and hence the contact between potentially susceptible and infected non-group members [4], [25], including aggressive interactions and bite wounding [26], [27].

Social disruption of remaining badger populations following culling has been shown to influence individual badger movements between social groups, with young females moving into depopulated setts [23], possibly to escape breeding suppression within their original group [28]. Dispersal by both sexes has been argued to maximise potential breeding opportunities [24], [28], [29], though extra-group mating may be common [30]. The epidemiological consequences of dispersal will depend on the contacts made between dispersers and members of the recipient social group [8], [11]. The movement of infected individuals as a mechanism for disease spread underpins much epidemiological theory [31], [32] as illustrated, for example, by foot and mouth disease [33], bovine tuberculosis in cattle [34] and possums [35], Severe Acute Respiratory Syndrome (SARS) [36] and prion disease in deer [37]. In the case of badgers, movement at both the individual and social group level have been identified as risk factors for bTB [38]. Behavioural heterogeneities and variability in contact rates between individuals are known to affect patterns of disease spread, particularly from studies of human sexually transmitted diseases, such as gonorrhoea [39] and, more recently HIV [9]. At the popualtion level, Rogers et al. [24] reported that bTB incidence in the Woodchester Park badger population was positively correlated with the rate of individual movement between social groups.

Evidence that badger culling operations can cause social perturbation has been reported from badger populations in Gloucestershire, UK [21], [23], [25], [40] and during the RBCT [22], [41], [42]. In all cases, the socio-spatial organisation of the populations was disrupted following culling, with, for example, group range overlap increasing [40]. These findings have raised the possibility that perturbation effects may have exacerbated disease spread within badger populations and contributed to the failure of reactive badger culling to control bTB in cattle [16], [21], [22], [42]–[47].

We report here on a field experiment carried out within the RBCT Triplet E (refered to as the Triplet E Experiment: TEE [21]) designed to examine perturbation effects following culling within the RBCT reactive strategy. The rationale was to compare a ‘treatment’ badger population, which was subjected to culling operations by the UK Government's Department for Environment, Food and Rural Affairs (Defra), with a control population which was not subjected to culling operations during the same period.

The following cascade of predictions arise from the perturbation hypothesis and from previous findings:

Badger culling influences the movement of individuals surviving within, and in the vicinity of, culled populations;

Changes in badger movement following culling will influence the epidemiology of bTB by affecting contact rates between individuals and consequential disease spread;

bTB within culled badger populations will spread more widely as a consequence of perturbation.

## Results

Over the four years of the study, a total of 663 badger captures with release was made in the culling (n = 481) and non-culling (n = 182) study areas, involving 423 individual animals. The overall rate of recapture was 29.7%, being approximately equal for males and females (28.3% and 30.1% respectively). Recapture rates were also consistent between study areas, being 30.1% in the culling area and 29.1% in the non-culling control area. Over the course of the study, the estimated overall density of badgers varied between 1.2 and 6.9 badgers per km^2^ in the culled population and between 3.9 and 7.1 badgers per km^2^ in the control population. Culling in the treatment study area took place in November 2002 (38 animals removed from 12 setts), January 2003 (six animals removed from three setts), and August 2003 (33 animals removed from 13 setts). The estimated population density in the treatment area fell to its lowest level of 1.2 badgers per km^2^ following the August 2003 cull, compared with approximately 6 badgers per km^2^ in the control site and approximately 5 badgers per km^2^ in the treatment site during the same season in the previous year. From our trapping records, the 77 badgers killed during culling operations amounted to between 34–43% of the badgers resident in the targeted social groups, based on the estimated number of animals within each group from our capture records.

### Prevalence and Distribution of bTB Infection

Of the 423 individuals captured during the study, 40 individuals were found to be positive for *M. bovis* (excreting) from one or more clinical samples. Of these animals with confirmed disease, eight were recaptured and none was found to excrete *M. bovis* intermittently (i.e. *M. bovis* detected in an earlier trapping, but not detected in subsequent trappings).

**Figure 1:**
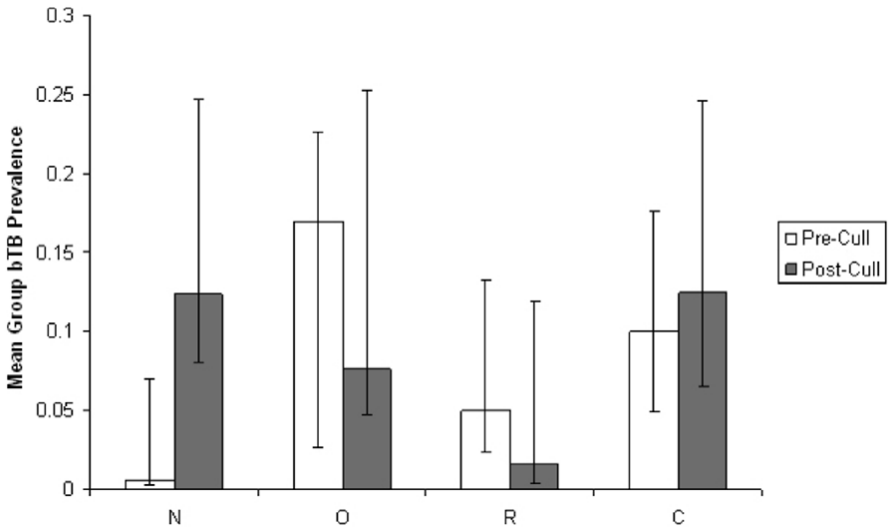
Mean bTB prevalence in Removed (R), Neighbouring (N), Other (O) and Control (C) badger social groups before and after culling. Error bars show 95% binomial confidence intervals.

There was no statistically significant difference in the detected prevalence of badgers excreting *M. bovis* between treatment and control sites (χ^2^ = 0.41; DF = 1; P = 0.52), with estimates of 8.1% and 7.1% respectively. However prevalence increased in both sites post-culling, from 5.2% to 15% in the treatment site, and from 2.7% to 15.9% in the control site (χ^2^ = 10.70; DF = 1; P<0.01). The effect of culling on the prevalence of detected *M. bovis* in badgers differed among social group types in the treatment area (Removed, Neighbouring and Other) following culling (Group Type×Cull: F_2,53_ = 4.35; P = 0.02), with the greatest increase being in Neighbouring groups: from 1.0% (sd = 4%) to 13.6% (sd = 12.3%) (fig. 1).

There was a statistically significant increase in detected *M. bovis* among cubs in the treatment population following culling, with only one excreting individual identified out of 101 cubs captured before culling (0.9%), and eight out of 76 cubs (10.5%) testing positive for bTB after culling (Two-tailed Fisher's Exact Test: P = 0.01). Of these eight cubs, four tested positive for *M. bovis* from urine samples, three from sputum and one from a faecal sample. No *M. bovis* was detected among the 47 bager cubs tested in the control population during this study.

### Individual Badger Home Ranges

Radio-collars were fitted to 50 (32 male and 18 female) and 20 (9 male and 11 female) adult badgers in the treatment and control study areas, respectively. From the culture of clinical samples, six of the collared individuals were found to be infected with bTB: four males and one female in the treatment; and one female in the control population. In total, 8,311 fixes were collected from tracked individuals over 239 nights. Radio-collars remained attached to badgers for an average of 230 days (sd = 54 days).

**Figure 2:**
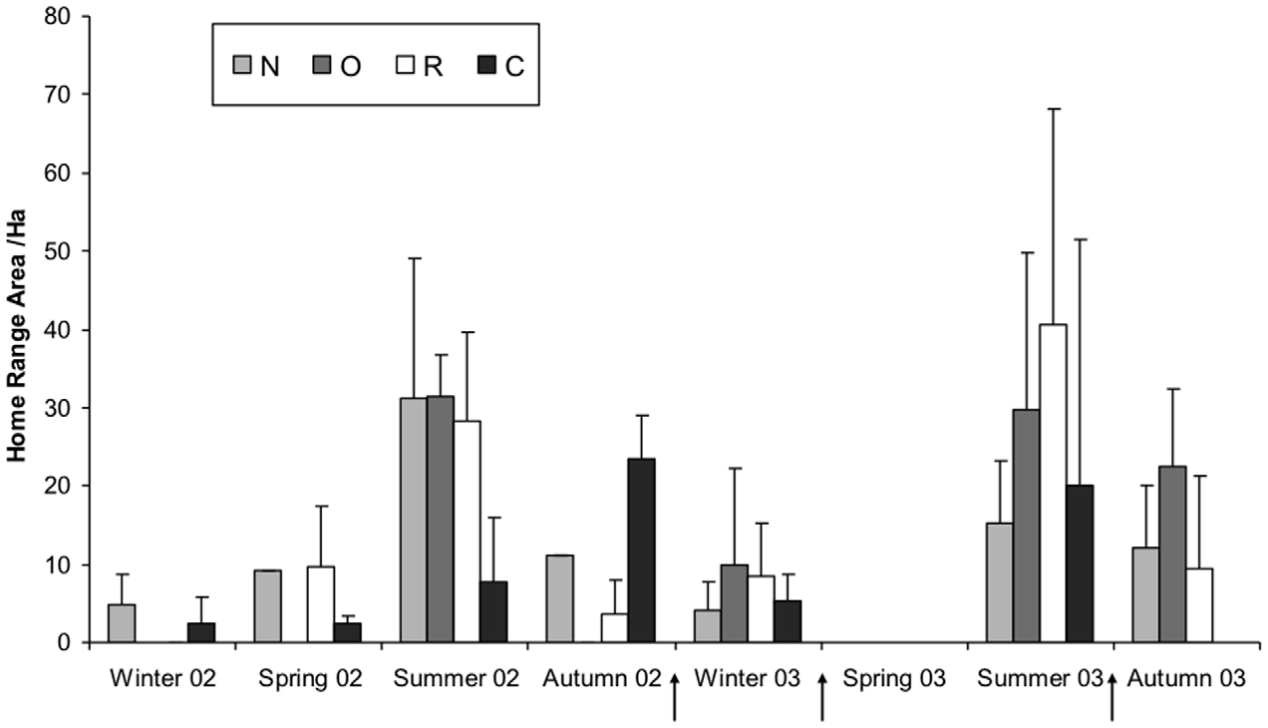
Mean range areas (95% minimum convex polygon) of individual badgers from Removed (R), Neighbouring (N), and Other (O) social groups in the treatment (culled) population, and Control (C) social groups (survey-only area). Arrows indicate the timing of badger removal operations. Error bar indicate upper 95% confidence intervals.

Home range size, as measured by 95% minimum convex polygons (MCP), varied seasonally, with maximum range (40.6 Ha) areas observed in summer in the treatment area and in autumn (23.4 Ha) in the control area (Season×Study area: F_3,81_ = 12.87 P<0.01). Within the treatment area the variation among cull phases was not consistent among group types (Group Type×Cull: F_4,40_ = 4.37, P<0.01, fig. 2). Home range size in Removed groups, tended to decrease through the culling phases (F_2,19_ = 6.63, P<0.01), from an average of 21.4 Ha (sd = 22.8 Ha) pre-culling, to 9.4 Ha (sd = 12.7 Ha) post-culling. However, size of summer range areas of badgers from Removed groups increased between the pre- and inter-culling phases during which they were measured, from 28.3 Ha (sd = 11.3 Ha) to 40.6 Ha (sd = 27.6 Ha). In Neighbouring groups, home range areas decreased between the pre- and inter-culling phases from 22.0 Ha (sd = 26.2 Ha) to 10.0 (sd = 11.3 Ha), remaining low post-culling (mean = 12.1 Ha; sd = 10.8 Ha), with this trend approaching statistical significance (F_2,16_ = 3.38, P = 0.06). There was an upward trend in home range size for badgers from Other and Control groups (F_2,6_ = 14.18, P<0.01).

The effect of culling phase on the size of badger home range core areas, based on 50% kernels, varied between study areas (Cull×Study Area: F_1,78_ = 10.33, P<0.01). Core areas in the treatment population tended to expand in response to culling, with increased variability, from a mean of 2.2 Ha (sd = 2.1 Ha) to 2.5 Ha (sd = 6.3 Ha).In the control population, core areas were smaller than in the treatment and decreased through the culling phases, from 1.2 Ha (sd = 1.4 Ha) to 0.8 Ha (sd = 0.6 Ha). Within the treatment population, however, there was no statistically significant difference in badger core area size between social group types with respect to culling (Cull×Group Type: F_4,40_ = 2.11, P = 0.10).

**Figure 3:**
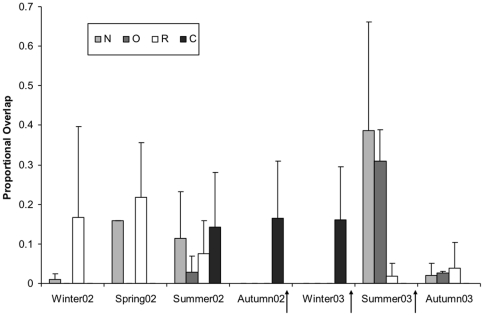
Proportional overlap between 95% MCP home ranges of badgers from different social groups for Removed (R), Neighbouring (N), and Other (O) social groups in the treatment (culled) population, and Control (C) social groups (survey-only area). Arrows indicate the timings of badger culling operations. Error bar indicate upper 95% confidence intervals. Overlaps in Autumn02 and Winter03 were zero for treatment groups (RNO), whilst no data were available for overlaps in Winter02 and Spring02 for O and C groups.

Within the treatment area, the effect of culling phase on proportional overlap between badger summer home ranges varied significantly among group types (Cull×Group Type: F_2,6_ = 5.48, P = 0.04: fig. 3), with an increase in inter-range overlap for Neighbouring groups (from 7.5 to 13.0 Ha on average) following culling, compared with Removed groups (from 8.2 to 10.8 Ha on average). Proportional overlap between home ranges in Control groups did not change significantly in relation to culling (F_1,17_ = 0.37, P = 0.56).

Similarly, the numbers of home ranges from different groups with which each badger overlapped in summer also varied among group types in the treatment population in response to culling (Cull×Group Type: F_2,6_ = 6.79, P = 0.03). Neighbouring and Other group overlaps increased following culling, from an average of 0.8 to 2.7 groups with which individuals affiliated, compared with a decline in Removed groups from approximately 0.8 to 0.2 on average. The number of overlaps appeared to remain relatively high in Other groups in autumn 2003, compared with 2002, when radio-tracking of six animals from five adjacent social groups revealed no home range overlaps. Numerical overlap between home ranges in Control groups did not change significantly in relation to culling (F_1,17_ = 0.06, P = 0.81).

### Inter-group Movement (IGM)

Daytime positioning revealed inter-group movements (IGM) by radio-collared badgers twice out of 891 below-ground positions between 2001 and 2003. Both of these were in the treatment population and were made by males to groups immediately neighbouring their original groups. One of these moves was made during the culling operation in November 2002 and the other six months after culling in July 2003.

**Figure 4:**
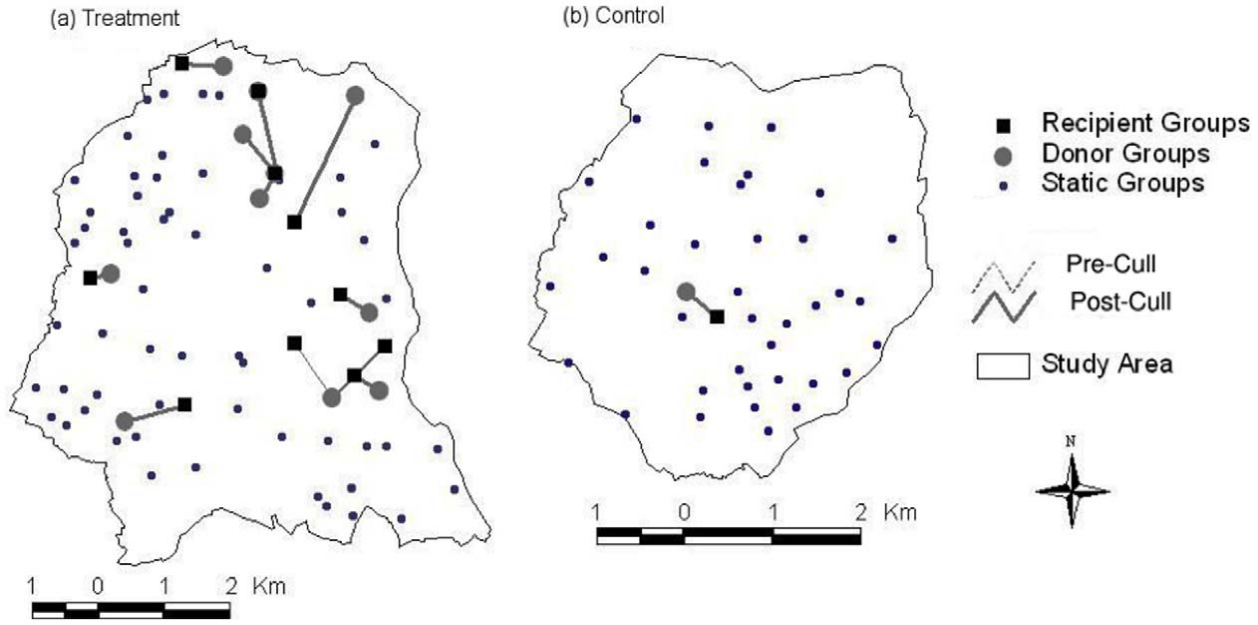
Inter-group movements (IGMs) of individual badgers from trapping records. IGMs in treatment (a) and control (b) study areas are shown before and after the Nov02/Jan03 culls. Symbols indicate the direction of movement from donor to recipient groups.

Out of a total of 663 trapping events (including 197 recaptures) in the treatment and control study areas, 12 instances of badgers moving between social groups (IGMs) were detected (11 individuals). Of these IGMs, nine (75%) occurred in the treatment population following the culls in Nov02/Jan03, and one occurred in the control study area (fig. 4). In total, 14 IGM events were identified by combining those detected from trapping and daytime positioning data. Badgers were more likely to move to Removed groups (χ^2^_1_ = 7.20; P<0.01), with nine post-culling IGMs occurring in the treatment population all being made to Removed groups. Five of these nine were from Neighbouring social groups, and four from Removed groups.

Within the treatment population, social group type influenced the probability of individuals moving from a group, with marginal statistical significance (Logistic Regression: W_1_ = 3.64; P = 0.056), with more movements observed from Removed (n = 5) and Neighbouring groups (n = 5) compared with Other groups (n = 1). The size of the donor social group influenced IGM probability (W_1_ = 4.37; P = 0.04), with animals being more likely to move from larger groups (average estimated group size with movement was 5.2 animals (sd = 2.1), compared with an average group size of 3.4 animals (sd = 1.9) where no movement was observed). IGM probability was not influenced by an individual's sex (W_1_<0.01; P = 0.95), with IGMs being made by seven females and six males; or by age (W_1_ = 1.32; P = 0.25), although nine of the movers were adults and only four were cubs. The adult sex ratio of the donor group was also not a significant predictor of IGM (W_1_ = 0.06; P = 0.80).

**Figure 5:**
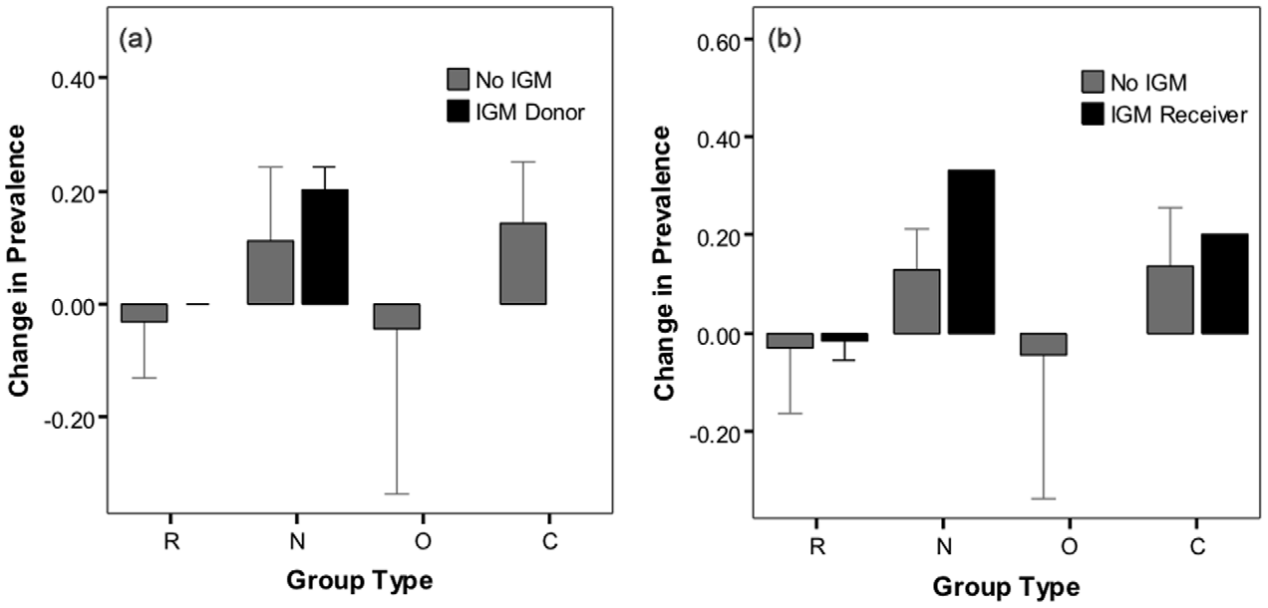
Mean change in bTB prevalence following culling by social group type (RNOC). Intergroup movement (IGM) from donor groups (a) and IGM receiver groups (b) are shown. Error bar indicate one side of 95% confidence intervals.

Overall there was a tendency for increases in the prevalence of detected *M. bovis* in Neighbouring social groups, associated with both IGM donors (fig. 5a) and recipients (fig. 5b), although neither relationship was statistically significant at the 5% α-level (F_5,44_ = 2.30, P = 0.06 and F_6,43_ = 1.97, P = 0.09 respectively). However, *M. bovis* excretion was not detected amongst the individuals identified as having made IGMs.

## Discussion

Information on the behavioural responses of badgers to culling is critical to furthering our understanding of the perturbation effect and the potential value of this management approach for the control of bTB infection in both badgers and cattle. In our study although inter-group movementss (IGMs) by badgers were rare, they increased significantly following culling. This usually involved the movement of animals into groups that had been subjected to culling either from other culled groups or their unculled immediate neighbours. The prevalence of detectable *M. bovis* infection from culture of clinical samples increased from 1% to 14% in Neighbouring groups following culling and this increase tended to be greater in groups involved in IGMs, whether as donors or receivers. The culture of clinical samples alone may prove insensitive, partly due to the potential for intermittent shedding of bacilli by badgers [48], [49], although this was not observed in this population [21]. Although the culture of clinical samples will have under-estimated true levels of M. bovis infection in badgers in this study, this effect will have been consistent and so does not invalidate comparison of relative levels of disease in space and time.

Within the treatment population, the relative sizes of badger core home range areas among group types were unchanged by culling. However, overall home range areas of individuals from Removed and Other groups, measured as MCP, increased significantly in response to culling, suggesting an increased range of movement by individuals from these groups. Individuals from Neighbouring groups increased the amount of home range overlap with individuals from surrounding groups, following culling, although the tendency was for their home range sizes to decrease through the culling phases. The increase in overlap is consistent with the observation from a separate study of the same population using bait-marking which showed that the group ranges of Removed and Neighbouring social groups also increased their overlap with adjacent social groups [21].

One simple prediction arising from the perturbation hypothesis is that the movement of infected animals from disturbed groups presents enhanced opportunities for disease transmission in the population. Our results were not consistent with this prediction, with instead there being a pervasive emergence of bTB across the badger population following culling [21]. While detectable bTB prevalence tended to increase in IGM receiver groups, no infection was detected in animals moving into these groups following culling, and prevalence in donor Neighbouring groups also tended to increase. It is plausible that animals detected making IGMs were infectious, but were not detected by the culture test because they were not shedding bacilli at the time of capture [50]. Furthermore, animals other than those captured will have been making IGMs, and some of them may have also been infectious. Nonetheless, the previously reported apparent absence of association between post-culling bTB infection and bite wounding, and the emergence of bTB infection in cubs [21] suggest that perturbation may act indirectly. For example, post-culling disturbance of the social structure of the badger population could potenitally cause sufficient stress to individuals to induce immunosuppression [21], [51], [52]. This could in turn result in the expression of latent disease already existing within badger social groups [53], or heightened susceptibility to infection from bacilli surviving in badger setts and elsewhere in the environment [54], [55]. In such circumstances, cubs may provide a useful barometer for the potential role of social stress in bTB infection, since their immune systems are immature and they consequently have lower resistance to disease [53], [56], [57].

Data on contact rates between individual badgers is scant, largely due to the practical difficulties of observing animals directly, particularly underground. In one undisturbed high density population, agonistic contacts between badgers were found to increase with density, as indicated by rates of bite wounding [27]. Aggressive contacts in the current study population increased in the wake of culling despite a reduction in population density [21]. In a separate study of three geographically distinct populations no consistent patterns of bite wounding were observed, suggesting that local conditions may be of particular importance [26]. In a small scales study of badger contact networks using proximity sensors, inter-group contacts were infrequent and found to be made by relatively few individuals [8].

The evidence from radio-tracking individual movements also demonstrated a widespread effect of culling. In Other groups there was a behavioural response to culling, with increases in home range size and overlap between individuals from Neighbouring and Other groups. The IGM rate increased between Neighbouring and Removed groups following culling, with the majority of movements being into Removed groups. IGMs in the control area, and prior to culling in the treatment area, were rare. Animals were more likely to move from larger groups, with no effect of sex or age. This contrasts with the observations of Tuyttens et al. [40] in North Nibley, Gloucestershire, where young females were found to move into setts in the year afer culling had taken place. Similarly, after a badger removal operation (BRO) near Woodchester in Gloucestershire, female badgers recolonised cleared setts in greater numbers than did males in the first six years following culling [25]. However, unlike the present study, both the North Nibley and Woodchester BROs involved virtually all badgers being removed from the targeted social groups. Culling operations in the present study however, removed approximately 40% of animals from targeted groups, with sufficient individuals remaining to produce cubs in the following breeding season [21]. Estimated capture rates of badgers from other triplet areas in the RBCT were measured as an index of the proportion of available traps that caught badgers [58], and varied from 5.4% to 11.8% (8.8% on average; Triplet E = 7.7%). These indices were not directly calibrated with badger population density and so comparison here is problematic. Culling was deemed to have affected a number of indirect indices of badger abundance in both proactive and, to a lesser degree, reactive treatments [58]. Differences in the intensity of culling may thus influence the movement behaviour of individuals from surrounding groups and hence the strength of any perturbation effect on population recovery and bTB epidemiology.

It is difficult to generalise about badger movement patterns [59], with evidence available to show that dispersal or excursions can be male-biased [24], [60], [61], female-biased [29], [62], or independent of gender, as observed here. It is similarly difficult to draw general conclusions on the existence of movement gradients from large to small groups suggested in the present study and in higher density, undisturbed populations for both males [24] and females [29]. Nonetheless, our observations provide further evidence of the enhanced movement of badgers associated with culling, the precise characteristics of which may be subject to local conditions. Although limited in its scope, being a sample from only one RBCT triplet, our study confirms and provides signifcant further details on the epidemiological patterns emerging after badger culling which are likely to be counter-productive for the control of transmission to cattle.

## Materials and Methods

### Study Areas

Study areas were located on the borders of Wiltshire, Somerset and South Gloucestershire in South-West England. The treatment badger population was positioned within the reactive culling area of the RBCT triplet E (51° 27′N 2° 25′W), occupying an area of 37.3 km^2^. The comparative control population, which was not subject to culling by Defra, occupied an 18.9 km^2^ area within the survey only area of triplet E (51° 28′N 2° 03′W). Both areas included mixed farming, with arable and livestock production, the latter being principally dairy cattle. The agricultural landscapes of both areas created mosaics of woodland, pasture and arable fields.

Both treatment and control study areas were surveyed for badger setts initially in autumn 2000. Setts were categorised as either active or inactive based on the condition of entrance holes and the freshness of badger sign in the vicinity. In total, 71 and 38 active setts were identified in the treatment and control study areas respectively, giving a density of approximately two setts km^−2^ in both areas.

### Culling Operations

Within the treatment study area, Defra performed three badger culling operations in November 2002 (in which 38 badgers were removed from 14 social groups), January 2003 (in which six badgers were removed from four social groups) and August 2003 (in which 33 badgers were removed from 13 social groups).

### Badger Live Capture and Collection of Clinical Samples

Badgers were captured at active setts using wire mesh cage traps, with a 2.5 cm mesh size, placed at or near to setts and baited using peanuts. Traps were set over two consecutive nights, following at least one week of pre-baiting. Trapping was carried out between September 2002 and August 2003, with study areas trapped during winter (January), spring/early summer (May/June), summer (August) and autumn (September, October and November).

Captured badgers were sedated using a combination of ketamine hydrochloride (100 mg/mL, Vetalar V, Pharmacia and Upjohn), medetomidine hydrochloride (1 mg/mL, Domitor, Pfizer) and butorphanol tartrate (10 mg/mL, Torbugesic, Fort Dodge Animal Health) by intramuscular injection at a ratio of 2∶1∶2 by volume respectively and a dose rate of approximately 0.2 mL/kg [63]. On first capture each individual was given a unique identifying tattoo on the belly [64]. Samples of sputum, urine, faeces and pus from wounds or open abscesses were taken from anaesthetised badgers, and were cultured to detect the presence of *Mycobacterium bovis* [65], the causative agent of bTB.

### Ranging Behaviour of Individuals

The home ranges of individual badgers were determined by radio-tracking, using radio-transmitters in the 173 MHz band, attached to a leather collar (BioTrack Ltd, Wareham, Dorset, UK). Adult badgers were selected for radio-collaring based on existing collars within the same social group, spatial proximity of collared animals from adjacent social groups, and sex. The objective was to obtain a wide distribution of several clusters of collared animals of approximately equal sex ratio. Clusters were initiated by the opportunistic capture of two more adults of different sex from a social group. On subsequent trapping occasions, adults captured from these social groups, and their immediate neighbours, were collared preferentially, until two animals of each sex had been collared from each social group. This event only occurred at two social groups; one in the treatment area and one in the control area. Clustering collared animals over three or four neighbouring social groups provided the ability to examine overlap and interactions between individuals from different groups. The wide distribution of these clusters minimised bias due to the locations of (at that time, unpredictable) reactive culling operations which might have subsequently taken place within the treatment study area. Radio-collars were also deployed opportunistically as the need arose when practicalities thwarted adherence to the decision strategy, such as a lack of candidate individuals from a particular social group.

Fixes were taken at a maximum interval of 15 minutes, decreasing to 5 minutes when badgers were moving. Observers radio-tracked individual focal animals at any one time preferentially, with fixes also established for other collared animals in the vicinity of the focal animal. Fixes were obtained using either Mariner (BioTrack Ltd, Wareham, Dorset, UK) or R-1000 (Communication Specialist Inc, Orange, CA, USA.) receivers with six-element Yagi-style directional antennae (BioTrack Ltd, Wareham, Dorset, UK). Positional data were recorded on 1∶10,000 scale maps or with global positioning system (GPS) receivers (Garmin eTrax 12-channel). Badger positions were established either visually, with the aid of image intensifying equipment or by triangulation.

Badger day-time sett locations were also established using radio-telemetery. Only badger presence could be determined, as failure to locate an animal at a particular sett did not necessarily demonstrate absence. Failure to locate a badger at either the sett at which it was captured or the sett to which it was last radio-tracked initiated a survey of all surrounding setts until either the badger had been located or no more setts remained to survey.

### Data Analysis

Following the protocol of Tuyttens et al. [23], [40], social groups were classified according to their use of setts and the proximity of those setts to culling operations. Within the treatment study area social groups were classified as Removed (R) if they were the target of culling, Neighbouring (N), if they were immediately adjacent to Removed groups, or Other (O), if they were adjacent to Neighbouring groups or beyond. All groups within the control area were classified as Control (C). Group type was included as a categoric fixed factor in analyses to alleviate any effect of spatial autocorrelation, since related social groups are pooled within the analyses.

#### Individual Ranges

Individual ranges were estimated from radio-tracking data using geographical information system software (ArcGIS 9.2: www.esri.com) and Ranges home range analysis software (www.anatrack.com). Total home ranges of badgers were defined as 95% minimum convex polygons (MCP) and core areas within badger home ranges were identified using 50% kernels [66]. A reference smoothing factor was applied during kernel estimation [67]. In order to preserve maximum biological information, positional fixes were not sub-sampled to reduce autocorrelation between successive fixes [68], [69].

Radio-tracking data were grouped into seasons: spring (Mar, Apr, May), summer (Jun, Jul, Aug), autumn (Sep, Oct, Nov), and winter (Dec, Jan, Feb) . Radio-tracking data were collected throughout the study, from Nov 2001 to Dec 2003, except during culling operations, at Defra's request, to avoid compromising RBCT results. Comparisons of individual movements before and after culling were adjusted for season, with spring and summer comparisons being made relative to culling in Nov 02 and Jan 03; winter comparisons being made relative to Nov 02 culling and autumn comparisons being made relative to all culling events.

#### Intergroup Movement (Dispersal)

Defining dispersal is difficult, and the approach used should depend on whether trapping or radio-tracking data are used. Trapping provides at most a ‘snap-shot’ of residency, with longer term data needed to distinguish short-term excursions from more permanent shifts in residency [61]. Residency may be defined usefully as an individual being trapped consistently within the same social group [24], [29]. An animal would thus be identified as a mover if it fulfilled the residency criterion at more than one social group. Here we used the term ‘inter-group movements’ (IGM), based on both trapping and radio tracking data. An IGM was defined as occurring when a badger was either captured at a sett belonging to a different group than that at which it was captured previously, or when it was located at a sett belonging to a different group by night-time radio-tracking or daytime positioning.

### Statistical Analysis

The SAS software was used for data analyses [70]. We used the SAS MIXED procedure where response variables were continuous (appropriate transformations were applied for adherence to model assumptions). The declaration of either social group or badger identity as random factors allowed for non-independence arising from multiple measures on the same social groups or the same badgers, respectively [71].

Home range MCP and core areas, were log-transformed (log10+1) to satisfy the condition of normality, as was numerical overlap between home ranges. Responses expressed as proportions were arcsine-square root transformed (proportional overlaps between home ranges and proportional change in bTB prevalence). We first fitted a model using the predictors study area (treatment or control), cull (pre-, between or post-culling), season and all two-way interactions. Separate models were defined for the treatment population alone, comparing differences between social group types (RNO). No valid model could include both study area (treatment or control) and group type, as group type was entirely confounded with study area.

The factors influencing the likelihood of an individual moving from one social group to another (IGM) were examined using logistic regression (SAS GENMOD procedure), with animal movement entered as a binary response. Donor social group type (RNO), sex ratio and estimated size were entered as covariates, along with the individual's sex and age (adult or cub).
